# Editorial: From Perception to Action: The Role of Auditory and Visual Information in Perceiving and Performing Complex Movements

**DOI:** 10.3389/fpsyg.2019.02696

**Published:** 2019-11-29

**Authors:** Mauro Murgia, Tiziano Agostini, Penny McCullagh

**Affiliations:** ^1^Department of Life Sciences, University of Trieste, Trieste, Italy; ^2^Department of Kinesiology, California State University East Bay, Hayward, CA, United States

**Keywords:** perception, action, complex movements, sport, rehabilitation

In this introduction to the Research Topic “From Perception to Action. The Role of Auditory and Visual Information in Perceiving and Performing Complex Movements,” we would like to thank all the contributors for their valuable efforts. In addition, we are extremely grateful to the reviewers for their constructive comments and for helping us to improve the overall quality of the articles of this special issue. Finally, we thank the staff of the editorial office for their advice in the management of the review process and for their technical support.

This article collection extends our knowledge on the influence of sensory information on the perception and execution of movements, with a special focus on movement-related auditory and visual information.

In recent years, various studies investigated auditory information in complex movements, in terms of how sounds can affect movement execution (e.g., Thaut et al., 2015; Pizzera et al., 2017; Bailey et al., 2018; Murgia et al., 2018) and of how biological motion perception is affected by sounds (e.g., Allerdissen et al., 2017; Camponogara et al., 2017; Murgia et al., 2017; Sors et al., 2018). In our Research Topic, we include several contributions both on perception and motor execution, using in some cases purely auditory stimuli or combining/comparing auditory and visual stimuli. Interestingly, the contributions to this line of research cover a relative wide range of domains (sports, rehabilitation, music, and dance), providing readers with a quite large overview of applications of these studies.

As for the visual modality, many studies investigated the role of visual information on the perception of other's movement, with applications in sport anticipation (Williams and Jackson, 2019), perceptual training (Abernethy et al., 2012), and interpersonal coordination dynamics (Travassos et al., 2011; Nalepka et al., 2017). In this special issue, we host contributions which provide new knowledge in these fields, showing empirical findings deriving from a quite large variety of paradigms and research methods (e.g., point-light displays, spatial and temporal occlusions, virtual reality, eye tracking).

The articles of this collection are grouped in four sections.

## Auditory Information in Sport, Exercise and Rehabilitation

The first contribution of this section is a review by Schaffert et al., describing the mutual influences between complex movement and sound. The authors critically analyze the studies on ecological sounds and movement sonification in sports and those on rhythmic auditory information and sonification in rehabilitation. The next two contributions address two methodological issues. The contribution by Schmitz et al. proposes a new method based on movement sonification for the rehabilitation of patients with stroke. In particular, the authors contribute a “Clinical study protocol article,” describing a protocol that provides auditory real-time feedback on upper limb movement, aimed at helping patients participating to a motor rehabilitation program after stroke. The work by Ghiselli et al. illustrates three clinical cases of children with congenital hearing impairment engaged in non-instrumental musical training. The authors describe this training and its effects on cognitive and motor skills, discussing the preliminary evidence of this method and its potential clinical relevance.

The last two contributions of this section are original research articles. The study by Ghai et al. investigates the effects of auditory feedback in real-time to facilitate knee proprioception. The authors provide empirical evidence that the use of auditory feedback improves the accuracy of knee re-positioning and that this effect can be modulated with step-wise transposition of frequency. The authors discuss the potential applications of their finding in rehabilitation settings. Conversely, the last work of this section—by Kreutz et al.—concerns the effects of loud music in sports, and in particular on ergometer exercise. The authors investigate the effects of electronic music, manipulating the intensity levels, and evaluated the ergometer performance, the perceived fatigue and the heart rate in university students with relatively high and low levels of training.

## Auditory and Visual Information In Motor Learning and Imagery

This section includes those contributions examining the effects of auditory and visual cues (either compared or combined) on movement or imagery. The study by Bienkiewicz et al. investigates whether auditory and visual cues regarding the kinematic of experts can enhance motor learning in golf, demonstrating that both auditory and visual cues can be beneficial for novices. Bläsing et al. focus on motor learning in dance. They compare visual cues and verbal instructions and show that the latter are more effective than the former, when learning dance movements. Finally, Yu et al. investigate the lower limb imagery alone or combined with visual or audiovisual stimuli, using neurophysiological measures. They find that the visual-auditory stimuli produce the most valuable effects, with important implications for motor learning and rehabilitation.

## Visual Information and Motor Expertise

This section starts with the contribution by Kurz and Munzert, who present a mini review on football penalty takers and eye movements. In particular, they analyze how experimental artificial conditions influence gaze behavior. The second contribution of this section is an original study by Vickers et al.. The authors investigated the role of quiet eye in basketball, and in particular they focused on the timing and the location of fixations, and on the effect of the defender on performance, in three-point shots. The next contribution—by Jackson et al.—further analyzes the role of visual perception in football. In this case, the authors used the spatial and temporal occlusion paradigms to investigate the ability to discriminate between genuine and deceptive actions, and examined the sensitivity to different sources of visual information of the opponent.

The third article of this section is by Bläsing and Sauzet and investigates the perception of action in the domain of dance. In particular, the authors analyze the participants' ability to recognize point-light displays of dance-like actions, previously performed by the same participants. The next article is by Marchal-Crespo et al. and deals with different training strategies to enhance motor learning. In their work, the authors focus on the learning process of a modified gait pattern, and compared the haptic error modulation and the visual error amplification strategies. Finally, this section ends with the contribution by Castañer et al., who study the laterality profile and the approach of young athletes on a novel perceptual-motor situation. In particular, they examine how the athletes use the limbs and investigated their spatial orientation.

## Interpersonal Coordination and Sensory Information

The last section of this special issue is dedicated to original studies on interpersonal coordination, interactions among actors, and perception of others' point of view. The first contribution of this section is by Hwang et al., who examine the social coupling between two individuals in a collaborative task. They manipulate the perceptual information available, by combining visual information with different types of auditory feedback. The next study by van Opstal et al. focuses on the investigation of interception, using a doubles-pong task. In particular, the authors study how teams intercept approaching balls, when teams are composed of two different level players. The third study of this section is by Meerhoff et al., who focus on collision avoidance. In their study, the authors examine the strategies of dyadic avoidance compared to triadic avoidance, and how locomotor interactions are influenced by the dynamics of a passable gap between two walkers. Finally, the last contribution of this section (and of the entire article collection) is by Cook et al.. In this study, the authors investigate how naturally produced virtual motion can affect postural regulation. Moreover, they study the response to different types of optical flow, which was produced by other individuals.

## Final Remarks

As editors, we are fully satisfied with this collection of articles and are convinced that most of them will have a high impact on research in this field. We hope that these works will stimulate new ideas, and contribute to the development of research on the mutual influences between auditory and visual perception and complex movements.

## Author Contributions

MM, TA, and PM contributed equally to the development of the outline of this editorial. MM wrote the first draft, which was revised and edited by TA and PM. All the authors approved the final version of the manuscript.

### Conflict of Interest

The authors declare that the research was conducted in the absence of any commercial or financial relationships that could be construed as a potential conflict of interest.
